# A Strong Impact of Genetic Background on Gut Microflora in Mice

**DOI:** 10.4061/2010/986046

**Published:** 2010-07-20

**Authors:** R. Steven Esworthy, David D. Smith, Fong-Fong Chu

**Affiliations:** ^1^Department of Cancer Biology, Beckman Research Institute of the City of Hope, 1500 Duarte Road, Duarte, CA 91010-3000, USA; ^2^Division of Information Sciences, Beckman Research Institute of the City of Hope, 1500 Duarte Road, Duarte, CA 91010-3000, USA

## Abstract

Genetic background affects susceptibility to ileocolitis in mice deficient in two intracellular glutathione peroxidases, GPx1 and GPx2. The C57BL/6 (B6) GPx1/2 double-knockout (DKO) mice have mild ileocolitis, and 129S1/Sv (129) DKO mice have severe inflammation. We used diet to modulate ileocolitis; a casein-based defined diet with AIN76A micronutrients (AIN) attenuates inflammation compared to conventional LabDiets. Because luminal microbiota induce DKO ileocolitis, we assessed bacterial composition with automated ribosomal intergenic-spacer analysis (ARISA) on cecal DNA. We found that mouse strain had the strongest impact on the composition of microbiota than diet and *GPx* genotypes. In comparing AIN and LabDiet, DKO mice were more resistant to change than the non-DKO or WT mice. However, supplementing yeast and inulin to AIN diet greatly altered microflora profiles in the DKO mice. From 129 DKO strictly, we found overgrowth of *Escherichia coli*. We conclude that genetic background predisposes mice to colonization of potentially pathogenic *E. coli*.

## 1. Introduction

Gut microbiota play an important role in several diseases including inflammatory bowel disease (IBD), type-1 diabetes, and obesity [1]. Recent metagenomic studies of the gut microbiota have shown that bacteria dominate the gut ecosystem [2]. Between 90 and 98% of bacteria, sampled either from gut-surface-adherent population or feces, belong to four bacterial phyla *Firmicutes*, *Bacteroidetes*, *Proteobacteria,* and *Actinobacteria* [3, 4]. Although there is a large variation in bacterial population in different individuals, the same bacterial phyla predominate in the stomach, small intestine, colon, and feces from the same individual [2, 5]. However, some IBD patient guts have decreased bacterial diversity with depletion of members of *Firmicutes *and *Bacteroidetes* [3, 4]. Since understanding gut microbiota may provide insight for IBD risk, pathogenesis, and treatment strategies, there is surprisingly little information on the microbiota information in mouse models of IBD.

While the metagenomic sequencing study on human fecal microbial genes has expanded the database of bacterial genomes deposited in the GenBank, the findings on the gut microbiota composition also confirm the results from methods based on bacterial 16S ribosomal RNA (rRNA) gene sequences [3–5]. Other noncultured PCR-based methods have been used to appraise gut microbial composition; these include automated ribosomal intergenic spacer analysis (ARISA) and terminal restriction fragment length polymorphisms [6, 7]. ARISA utilizes conserved 16S and 23S rRNA gene sequences coupled with variability in the length of the intergenic spacer to discriminate among bacterial species. The PCR products are separated by an automated capillary electrophoresis system with single-nucleotide resolution and detected by a sensitive laser beam to produce an electropherogram.

ARISA has been used as a crude microbe assay. Metagenomic study has estimated that each individual harbors at least 160 bacterial species and entire cohort harbors between 1,000 and 1,150 prevalent bacterial species [4]. A single ARISA primer set on fecal samples only yields 20–30 consensus products and 100 across all subjects [7–9]. Nevertheless, because ARISA generates a highly reproducible microbiota profile with conventional instrumentation, we applied this method to assess cecal microbiota in a mouse IBD model.

We have generated a mouse IBD model by disruption of two genes encoding for two intracellular glutathione peroxidases, GPx1 and GPx2 [10, 11]. These GPx1/2-double knockout (DKO) mice (on a mixed C57BL/6 and 129S1/Sv genetic background) have microflora-dependent ileocolitis, since germ-free mice do not have inflammation [11]. Similar to other mouse IBD models, genetic background has a profound effect on disease severity in GPx1/2-DKO mice. B6 DKO mice have mild ileocolitis, the mixed-strain B6; 129 DKO mice have more severe disease [12], and 129 DKO mice have the most severe inflammation (from this study). Since B6 and 129 strains may have different innate immune responses, which can modulate microflora community [2, 13], we hypothesized that these two strains of mice also have different gut microbiota. 

In addition to genetics, diet also can modulate IBD. Patients with Crohn's disease (CD) can be managed by prescribed diets, which are almost as efficacious as anti-inflammatory corticosteroids [14–16]. For pediatric CD patients, the enteral nutrition is preferred to corticosteroids to avoid adverse effects in European countries [17]. The major impact of enteral nutrition may rely on changes in gut microbiota [16]. Thus, we also tested whether diet impacts on the ileocolitis and microflora in the DKO mice.

In this manuscript, we analyzed the dietary effect on mouse IBD on both B6 and 129 genetic backgrounds. Based on the current knowledge on gut microbiota in IBD patients, we tested whether mouse genetic background, inflammation (DKO genotype), and diet affected gut microbiota.

## 2. Materials and Methods

### 2.1. Mice and Diets

Generation of GPx1/2-DKO mice on the C57BL/6J (B6) × 129S1/SvimJ (129) background (B6;129) was described previously [10]. B6 colony was obtained after backcrossing B6;129 mice to B6 for 8 generations. N5 and N10 129 colonies were from B6;129 mice backcrossing to 129 strain for 5 and 10 generations, respectively. Mice were fed either commercial chows (LabDiet, Richmond, IN) or casein-based defined diets with AIN76A micronutrients (AIN; Harland-Teklad, Madison, WI) (Table 1). As specified in the experiments, some AIN diets were supplemented with brewers' yeast or inulin (Oliggo-Fiber Inulin, a gift from Cargill Inc., Minneapolis, MN). 

When on commercial chows, breeders were maintained on a high-fat LabDiet, and pups weaned to a low-fat LabDiet at 22 days of age. When on AIN diets, breeders had 10% corn oil (CO) and pups had 5% CO. Morbidity describes wasting mice, which were likely to die in the next 24–48 hours, or with poor health indicated by low body weight, no weight gain and diarrhea, and unlikely to recover. When describing diet effects on the pups before weaning, the diet refers to the breeder diet. All experiments performed on these mice were approved by City of Hope IUCUC.

### 2.2. Histology

**Figure 1:**
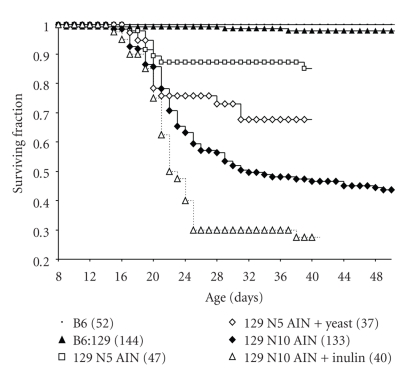
Kaplan-Meier curves showing the effect of genetic background and diet on DKO mouse survival. All mice are on AIN base diets. Monitored from 8 days of age, the surviving fraction excludes dead and culled, moribund mice. The numbers in parenthesis are the number of mice in each group. The data for yeast are pools both 1 and 10% as no difference could be distinguished. There are log rank differences for all interstrain comparisons on AIN diet: *P* < .0001. 129 N5, yeast-supplemented AIN (AIN + yeast) diet versus AIN; *P* = .0087. 129 N10, inulin-supplemented AIN (Ain + inulin) diet versus AIN; *P* = .0003.

**Figure 2:**
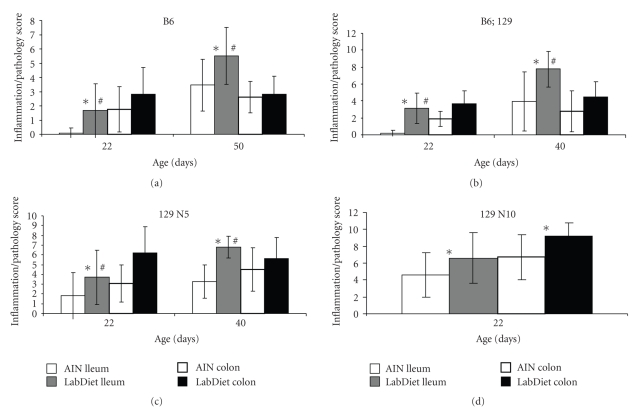
Effect of diet on ileum and colon histology at weaning and peak pathology by strain in GPx1/2-DKO mice. Mice on LabDiet or AIN diet were analyzed at 22 days of age or upon signs of morbidity, and at the peak of ileitis, which is 40 days for B6;129 or 129 N5 and 50 days for B6. 129 N10 mice were only analyzed at 22 days of age due to high morbidity. The AIN diet alleviated ileitis significantly in all strains at both 22 days of age and at peak inflammation (the “∗” sign indicates *P* < .011). The ileal inflammation/pathology scores increased significantly from 22 days to peak of inflammation in all strains (^#^ *P* < .034). A diet-associated difference in colitis was only observed in 22-day-old 129 N10 pups (*P < .001).

**Figure 3:**
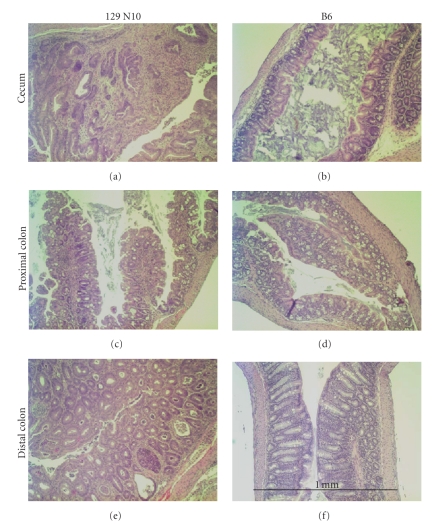
Representative histopathology in the colon of 129 N10 and B6 mice on AIN at 22 days of age. Slides were stained with hematoxylin and eosin. By 22 days of age, 90% of 129 N10 DKO mice have disease signs of diarrhea and many are lethargic. B6 DKO mice are without these signs. This is correlated with histopathology in the cecum and colons of these mice. 129 mice show inflammation and distortion of the cecum and distal colon with frequent crypt abscesses in the distal colon. The inflammation frequently skips the proximal colon (shown here). In severe cases, where lethargy has progressed to wasting, the proximal colon can be involved. The cecum and colon of B6 mice show mucin depletion and mild distortion due to apoptosis and proliferation.

Distal ileum and the entire colon were processed for histopathology analysis. Tissues were scored for inflammation and pathology in a blinded fashion using a 14-point system described previously [11]. Scoring includes lymphocytes and neutrophils infiltration (0–3 points), ileal Paneth cell or colonic goblet cell degranulation (0–2 points), epithelium reactivity, including crypt distortion (0–3 points), inflammatory foci (0–3 points), and apoptotic figures (0–3 points). The threshold for inflammation corresponds to a score of 6–7 [18].

### 2.3. Microbiota Census with Noncultured ARISA and Culture Methods

Cecal microflora were characterized in 22-day-old pups or younger (16- to 21-day-old) sick mice when morbidity criteria dictated. 

For noncultured ARISA, DNA was isolated from mouse cecal contents in 1 mL TE buffer (10 mM Tris-HCl, 1 mM EDTA, pH 8.0), 0.15 mL phenol, and 0.2 g of 1 mm Zirconia/Silica beads (BioSpec Products, Inc., Bartlesville, OK) using a minibead-beater (BioSpec Products, Inc.) [19]. Approximately 150–300 *μ*g DNA was extracted from the cecal contents of each mouse. The ribosomal integenic DNA was amplified by PCR using a primer set of ITSF (5′-GTCGTAACAAGGTAGCCGTA-3′) and ITSReub (Hex-5′-GCCAAGGCATCCACC-3′) as described [6]. The Hex-tagged fluorescent reverse primer is used to identify the products on the DNA sequencing instrument. One *μ*L of 25 *μ*L reaction mixture was analyzed with a capillary DNA analyzer (Hitachi AB model 3730) along with Genescan1000-ROX standard (Applied Biosystems; City of Hope Sequencing Core). To identify the DNA amplicons, the rest of PCR products were separated in agarose gels, and major DNA bands were excised and cloned into dT-tailed pCR2.1 (Invitrogen) and sequenced. Cloning total PCR products without gel separation only yielded one new sequence. DNA sequence identified was determined by BLAST (http://www.ncbi.nlm.nih.gov/). 

The cecal content was also cultured under aerobic and microaerobic conditions. The cecal contents were collected individually; the volume was measured and diluted with 10 volumes of sterile phosphate-buffered saline (PBS). Each sample was diluted 10,000 times the original sample volume with PBS and then plated with the original volume on two Luria-Bertani (LB) plates. One plate was incubated for 24 hours in aerobic condition, the other for 6-7 days under microaerobic conditions, both at 37°C [20]. For microaerobic culture, plates were placed in a GasPak (Becton Dickerson, Cockeysville, MD) with an activated Anaerocult A insert (EM SCIENCE, Gibbstown, NJ). The assembly was purged with CO_2_. After counting the colonies, up to 10 colonies from each plate were collected and DNA was amplified using ARISA primers. The PCR products were resolved in 1.3% agarose gels and DNA fragments were extracted with Qiagen Gel Extraction Kit and sequenced with the ARISA primers.

### 2.4. Statistics

**Figure 4:**
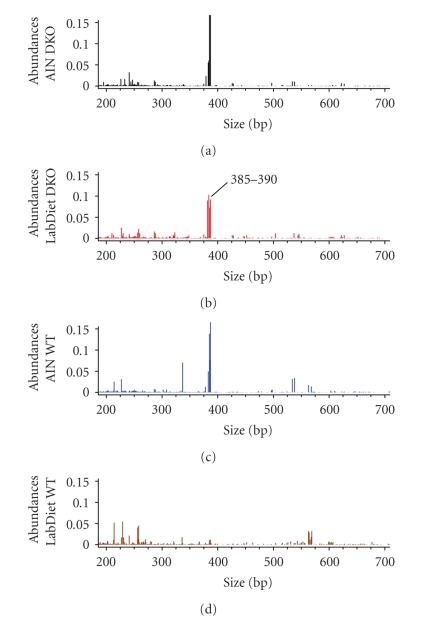
ARISA analysis of DNA from cecal contents of B6 on LabDiet or AIN diet. DNA amplified using primers corresponding to 16S and 23S ribosomal genes and oriented to contain the ribosomal intergenic spacer regions was resolved with a capillary DNA analyzer. The *x*-axis shows the size of the DNA peak, and *y*-axis shows the relative intensity of each peak. Each panel represents the average result of 6 mice.

For comparing time-to-event endpoints, such as survival time on different diets, the results were plotted with Kaplan-Meier curves and analyzed with the log-rank test. Analysis of variance (ANOVA) was used to compare the means ± standard deviations (SDs). To compare pair-wise diets versus a control, Dunnett's correction was made for multiple testing and the Dunnett-corrected *P*-values were applied. Each ARISA data panel represents results pooled from 6 to 21 mice analyzed individually. The electropherograms were digitized, and the results for each group were averaged using the statistical programming language R [21, 22]. The data were cleaned and passed to the Ribosort package created for R by Scallan et al. [23]. Ribosort detects and classifies peak-generating fragments in ARISA data with a two-pass algorithm [24]. Output from Ribosort contains information on the ribotype (represented by a specific size of PCR product) abundances, ribotype proportions, and sequencer detections. A Euclidean discriminant test was applied to the final step in ARISA data set analysis. Finally, a Czekanowski similarity index was run for pair-wise comparisons from all panels in the ARISA Figures 4, 6, and 8. The quantitative version of the Czekanowski similarity index is defined as 2*W*/(*A* + *B*), where *A* and *B* are the abundance of species in two given sample conditions and *W* is the number of species shared in the two samples. A convention for interpreting the Czekanowski similarity index is as follows: index between 0.5 to 0.75 indicates similar abundances; index between 0.25 to 0.5 indicates different abundances; index between 0 to 0.25 indicates very different abundances.

## 3. Results

### 3.1. Genetic Background and Diet Had a Profound Effect on GPx1/2-DKO Morbidity

Mouse strain background has a big effect on morbidity outcome in GPx1/2-DKO mice (Figure 1). As we noted before the strain difference in survival on the conventional LabDiet, here we report the same genetic effect on mouse survival on the AIN diet. The AIN diet, formulated to mimic LabDiet for calories, macro- and micronutrients, maintained better health than LabDiets for the DKO mice.

B6 GPx1/2-DKO mice maintained fairly good health on either a LabDiet or AIN diet. The surviving fraction of B6 DKO mice at 45 days on the AIN is 100%, which is virtually the same as mice on LabDiet with 98% survival compared at 45 days of age (Figure 1 and data not shown). The B6;129 mixed-strain DKO mice had marginal health, with 97% survival on AIN diet compared to 85% on LabDiet at 45 days of age. As expected, the 129 DKO mice had poor health. Eighty-five % of 129 N5 DKO on AIN diet survived 40 days when only 65% survived on LabDiet. Only 47% of 129 N10 on AIN diet survived 40 days when merely 5% of 129 N10 on LabDiet made it (*P* < .0001, Log rank; differences for strains between AIN diet and LabDiet, except B6). 

For 129 DKO mice, the early morbidity is associated with colitis. The postweaning morbidity in B6 and B6;129 DKO mice is correlated with later developing ileitis and rarely involves diarrhea. Disease in the 129 N5 and N10 DKO starts before weaning based on symptoms of wet tail and runting as early as 11 days of age on AIN diet.

### 3.2. Morbidity of GPx1/2-DKO Mice Is Correlated with Gut Inflammation

Morbidity at any time appears to be reflection of acute inflammation, reflected in the inflammation/pathology scores of 6 or greater [18]. B6 DKO mice had mean ileal pathology score around 5.5 at 50 days of age, the peak of inflammation in this strain (Figure 2). 129 N10 DKO mice had mean ileal and colonic pathology scores of 6.5 and 9, respectively, at 22 days of age. The pathology scores correlate with morbidity. Typical histopathology in 129 at 22 days of age showed acute inflammation in the cecum and distal colon with frequent skipping or less severe inflammation in the proximal colon (Figure 3). In morbid mice, disease often extended into the proximal colon. By contrast, B6 mice had almost no pathology in the cecum, proximal, and distal colon.

Diet significantly modified ileitis severity in all genetic backgrounds, that is, B6, B6;129, as well as 129 N5 and N10 DKO mice (Figure 2). Only in 129 strains was a dietary effect on colitis observed. Non-DKO mice did not have gut pathology.

### 3.3. Yeast and Inulin Supplementations to AIN Diet Increased Morbidity on 129 N5 and N10 DKO Mice

Since LabDiet has 1% brewer's yeast, and yeast antigens are associated with some CD, we tested its effect in 129 N5 DKO mice [25]. Feeding 129 N5 DKO mice with AIN diet supplemented with 1% and 10% brewer's yeast increased their morbidity (*P* = .0087) (Figure 1 shows pooled, no difference 1% versus 10%). The morbidity curve on yeast-containing AIN diet resembles that obtained with LabDiet (data not shown). Although the pups experienced more pronounced diarrhea, the pathology score was not elevated in the N5 DKO mice on the yeast-containing diet. The median colon pathology score was 3 at 22 days of age on both diets (*P* = .58), and 4.5 versus 4 for mice on yeast-containing AIN versus AIN diets at 40 days of age (*P* = .9; Mann-Whitney test). Thus, yeast-supplementation had an adverse effect on the DKO mice by increasing morbidity through intensifying diarrhea without exacerbating colitis.

Inulin, a nondigestible fructooligosaccharide is a food fiber with prebiotic properties, which may have prophylactic or therapeutic potential for IBD [1, 26]. We supplemented 5% inulin in AIN diet to 129 N10 DKO mice on the premise that it may retard the morbidity by fostering the colonization of probiotic microflora. Disappointedly, supplementing 5% inulin to AIN diet increased morbidity in 129 DKO pups (Figure 1; *P* = .0003). However, inulin did not affect colon inflammation; both diets yielded a median disease score of 7, *P* = .28 (Mann-Whitney test). Thus, inulin also had an adverse effect on DKO mice by increasing morbidity without affecting colitis.

### 3.4. Diet Modified Cecal Microbiota Content in Wild-Type (WT) B6 but Not DKO Mice

Since diet can alter gut microflora [16], and microflora are essential for IBD, we used ARISA to profile microbiota in these mice. We chose cecal content to analyze microbiota for the following reasons. First, the cecum is a disease site in GPx1/2-DKO mice. Severity of typhlocolitis is correlated with colitis in the 129 strain (*R* ^2^ = 0.63). Although the cecum has milder pathology than the colon (with the median disease score of 6 in the cecum and 8 in the colon of 129 N10 DKO, pooling AIN and LabDiet scores), the cecum was the major disease site in about 15% of mice. Second, unlike the colon, the cecum is rarely empty even in very sick mice. Third, little variability of microbiota is detected from six major subdivisions of healthy human colon: cecum, ascending colon, transverse colon, descending colon, sigmoid colon, rectum, and feces from the same person [5].

**Figure 5:**
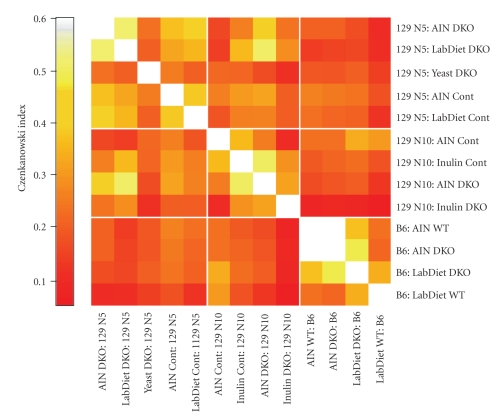
Heat map of Czekanowski similarity indexes of the ARISA profiles from Figures 4, 6, and 8. An index of 0.6 (white) is moderate similarity and an index of 0 (dark orange) is most distinct. “Cont” means the non-DKO sibs control of 129 DKO mice.

The microbiota profiles on B6 DKO and WT mice were determined individually on either LabDiet or AIN diet, and a combined profile was generated for each group of six mice by the Ribosort package programs (Figure 4). Each electropherogram was checked against the combined panel to confirm that the dominant DNA ribotypes in the panel were a consensus feature of the group. We used Czekanowski similarity index to compare the ribotype profiles between different mouse groups (Figure 5). This index is based on both presence and abundance of ribotypes without the bias of focusing on dominant ribotypes. Similar profiles were found in B6 DKO mice on either AIN or LabDiet and B6 WT mice on the AIN diet. B6 WT on LabDiet had the most distinct ribotype profile with dramatically diminished PCR products of 385–390 bp, which were prominent in the other 3 groups. The 385–390 bp products were sequenced and identified as either *Lactobacillus* species or *Bacteroides sp*. (Table 2). This result supports the notion that DKO mice have diminished diversity and thus more resistant to diet-associated alteration of microbiota.

### 3.5. Genetic Background and Yeast Supplementation Had a Dramatic Impact on Cecal Microbiota Studied in B6 and 129 N5 Groups

**Figure 6:**
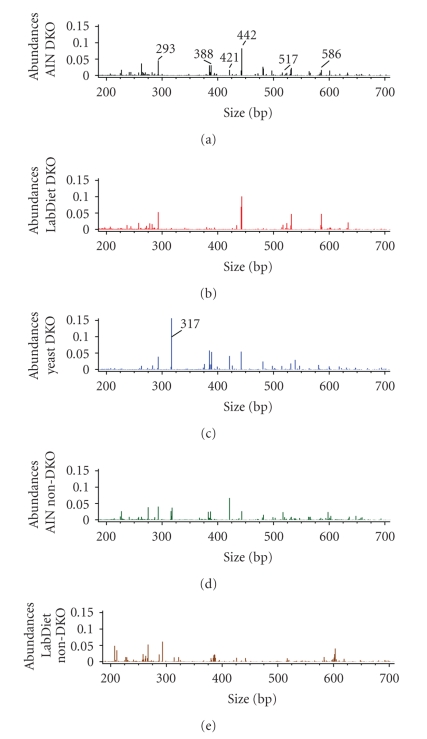
ARISA analysis of DNA from cecal contents of 129 N5. This DKO colony was established by backcrossing the mixed strain B6;129 colony to 129Sv/J for 5 generations. The plots were generated from 15 DKO mice on AIN diet (AINDKO), 18 DKO mice on LabDiet (LabDietDKO), 7 DKO mice on AIN diet supplemented with 1% yeast (Yeast DKO), 13 non-DKO mice on AIN diet (AIN non-DKO), and 13 non-DKO mice on LabDiet (LabDiet non-DKO). Non-DKO mice were the sibs of DKO mice, heterozygous for *Gpx1* and/or *Gpx2* (thus no inflammation). DKO mice on LabDiet did not have the distinctive peaks at 385–390 bp, 421 bp, 481 bp, and 498 bp found in other groups. DKO mice on the yeast-containing diet had a similar profile as AIN-fed mice, except having a prominent 317 bp peak. The 442 bp ribotype found in these 129 N5 DKO mice had lower abundance in the non-DKO sibs on both LabDiet and AIN diets.

We then compared ARISA profiles in 129 N5 DKO mice and their non-DKO littermates (heterozygous at *Gpx1* and/or *Gpx2*) on LabDiet, AIN diet, or yeast-supplemented AIN diet. Only minor differences were detected between each group of 7–18 mice, except the DKO mice on yeast-supplemented diet (Figure 6). Comparison by Czekanowski similarity index of ribotype profiles between different groups of 129 N5 mice indicated the most distinct profile was produced in the yeast-supplemented DKO mice (Figure 5). However, when comparing between B6 and 129 strains on either the same diet or the same GPx genotype, there is a striking difference in the ribotype profiles.

Noticeably, the prominent 385–390 bp products in B6 DKO were nearly nonexistent in the 129 N5 DKO mice on the LabDiet. The 442 bp ribotype was more prominent in 129 N5 DKO mice than their non-DKO littermates and was not detected in B6 groups. The 129 N5 DKO mice on the yeast-containing AIN diet produced a dominant ribotype at 317 bp and enhanced 385–390 bp ribotypes compared to the ARISA profile of 125 N5 DKO mice on the AIN diet (Figure 6). The 317 and 385–390 bp ribotypes were matched to *Lactobacillus sp*. or *Bacteroides sp*.

### 3.6. Association of the 442 bp DNA with Inflammation in the N5 129 DKO Mice

**Figure 7:**
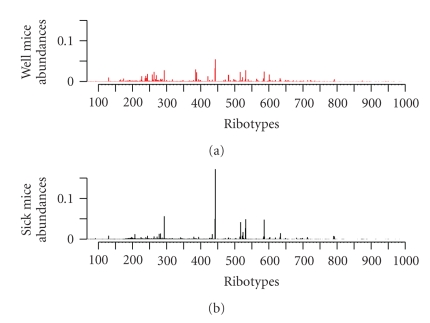
ARISA analysis of DNA from cecal contents of 129 N5 DKO mice stratified by the disease severity. Seven of 18 DKO mice on LabDiet, 13 of 14 DKO mice on AIN diet, and 9 of 16 DKO mice on AIN diet with daily contact of soiled bedding from LabDiet group were combined in the well group (29 mice) based on pathology. The other 19 DKO mice were stratified in the sick group. The median colon pathology score for well mice was 3 (range of 0–5) and 6 for sick mice (range of 6–11).

Since the microbiota profiles from the N5 129 strain mice were very similar, we stratified the DKO mice into sick versus well groups, regardless of the diet (Figure 7). In this comparison, the “sick” group had fewer ribotypes and a more prominent 442 ribotype. 

To identify the ARISA DNA products, we have performed sequence analysis on 9 different sizes of DNA PCR products, which matched to 3 bacterial phylum, *Firmicutes*, *Bacteroides,* and *Proteobacteri* (Table 2). Since multiple ribosomal intergenic spacers exist in each bacterium, some of the PCR products (such as the 442, 517, and 710 bp) belong to the same bacterium (*Escherichia coli, Shigella*, or *Salmonella*) from **γ*-Proterobacteri* phylum. As expected, none of the clones were matched to the mouse genome [6, 7].

### 3.7. Inulin Had an Opposite Effect on Cecal Microbiota in 129 N10 DKO and Non-DKO Control Mice

**Figure 8:**
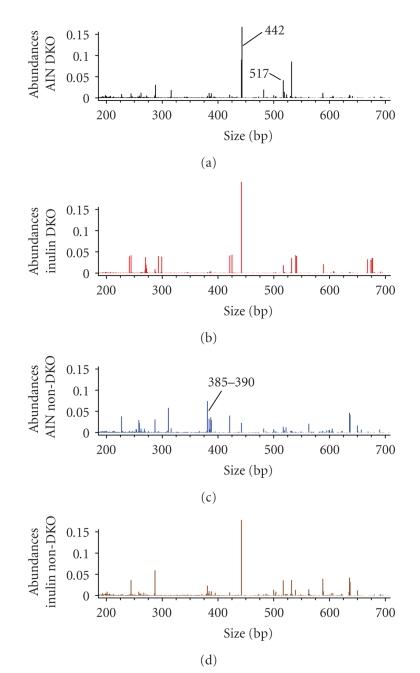
ARISA analysis of DNA from cecal contents of 129 N10. This DKO colony was established by backcrossing the mixed strain B6;129 colony to 129Sv/J for 10 generations. The group sizes are 21 DKO mice on AIN diet, 10 non-DKO mice on inulin-supplemented AIN diet, 7 non-DKO mice on AIN diet, and 15 DKO mice on inulin-supplemented AIN diet. The non-DKO mice are heterozygous DKO with 2 or 3 WT alleles for combined *Gpx1 *and *Gpx2*.

The ARISA profiles of the cecal bacterial content from the inulin-supplemented 129 N10 DKO showed increase of 12 ribotypes (240, 246, 270, 290, 295, 421, 425, 540, 542, 645, 650, 652–655 bp) (Figure 8). However, the prominent putative *E. coli* ribotype, 442 bp, in the AIN-fed DKO mice remained predominant. In the control non-DKO (with at least one WT allele for both *GPx1* and *GPx2* genes) mice, inulin supplementation caused reduction of many ribotypes. Czekanowski similarity index shows that the diversity of microbiota in inulin-supplemented 129 N10 DKO is most different from that in 129 N10 control non-DKO mice on AIN diet (Figure 5). Unexpectedly, the inulin-fed 129 N10 control mice had similar microbiota as 129 N10 DKO mice on the AIN diet. 

Comparison of Czekanowski similarity index between 129 N10 groups with 129 N5 and B6 groups shows that the 129 N10 had a more different microbiota profile from B6 groups than 129 N5 groups (Figure 5). Yeast-fed 129 N5 DKO mice had distinct microbiota from other groups. 129 N10 DKO on AIN diet had a similar and simplified ribotype profile as 129 N5 DKO on either LabDiet or AIN diet. The more diversified ribotype profiles present in 129 N10 non-DKO control mice on the AIN diet and 129 N10 DKO on inulin-AIN were also more distinct from those in 129 N5 groups.

### 3.8. Overgrowth of E. coli in the Cecum of 129 DKO Mice but Not 129 Non-DKO and B6 DKO or WT Mice

We cultured cecal contents under aerobic and microaerobic conditions to verify the identity of ribotypes obtained from the noncultured method and to estimate levels of *E. coli*. The putative *E. coli* colonies were identified by PCR with the ARISA primers, resolving of the dominant 442 and 517 bp products in agarose gels, and BLAST alignment of the sequences obtained from the PCR products. When the PCR was performed side-by-side with DH5*α*, a bioengineered strain of *E. coli*, the same products were obtained, confirmed by sizing and sequencing (data not shown). These colonies were identified as *E. coli* by the Microbiology Laboratory at City of Hope. Thus, the 442 bp PCR product is most likely indicative of *E. coli*. 

**Figure 9:**
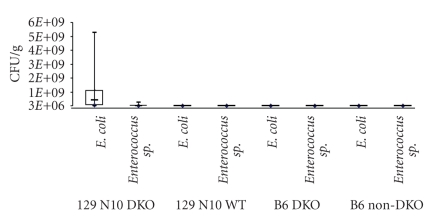
Box and whisker plots of the levels of cecal *E. coli* (large colonies on LB plates under aerobic conditions) and *Enterococcus sp*. (small colonies on LB plates; *E. faecalis, E. hirae, *or *E. gallinarum*) for mice on AIN diet. Bacteria counts are based on colony forming units per gram of cecal contents (CFU/g) at 24 hours, 37°C. Representative colonies were stabbed with a micropipet tip and transferred to ARISA PCR mix. The PCR products were resolved on agarose gels for comparison to laboratory *E. coli *and the PCR products were further isolated and sequenced for final identification of species.

The prediction that the 129 N10 DKO mice would show overgrowth of *E. coli *was substantiated by counts of cultures from cecal contents on LB plates (Figure 9). *E. coli *was detected in 28 of 31 mice on AIN diet at an average of ~1 × 10^9^ CFU/g cecal contents. On LabDiet, 6 of 6 DKO mice had detectable *E. coli* at an average ~1.5 × 10^9^ CFU/g. WT mice had average *E. coli* levels of ~1 × 10^6^ CFU/g, detected in 3 of 12 mice on AIN diet, which are at the lower limit of detection.

The 129 N5 cohort was not available for these follow-up studies; so their descendents at N7 were studied. *E. coli* colonies were isolated from 129 N7 DKO on either LabDiet (2 of 2 mice) or AIN diet (3 of 8 mice) cultured under both aerobic and microaerobic conditions at an average ~2 × 10^8^ CFU/g. No putative *E. coli* colonies were obtained from 129 N7 non-DKO mice (0 of 7 mice). These include 2 non-DKO siblings on LabDiet and 5 non-DKO 129 mice on AIN diet under either aerobic or microaerobic conditions.

Several other species of commensal bacteria were also isolated from 129 N7-N10 DKO mice. Under aerobic condition, *Staphylococcus* and* Enterococcus sp. *were cultured from two 129 N7 DKO mice (one well, one sick) on the AIN diet. No *E. coli *was detected in these two 129 DKO mice. The anaerobic plates from 5 of 8 129 samples yielded *Lactobacillus*, which also matched to a rat gut-uncultured bacterium, DR6-87 [28]. 

No *E. coli* was isolated from B6 DKO (*n* = 5) or WT mice (*n* = 7) on the AIN diet. On the LabDiet, *E. coli* was detected in 3 of 14 B6 DKO mice at an average level of ~2 × 10^7^ CFU/g. Under aerobic conditions, *Staphylococcus* was identified from 2 B6 DKO mice on the LabDiet and *Enterococcus hirae* from 1 mouse on the LabDiet. However, all mice produced *Lactobacillus sp*. (DR6-87) on microaerobic plates. *E. faecalis *and *Bifidobacterium* were isolated from one DKO mouse each on the LabDiet and the AIN diet, respectively.

## 4. Discussion

The genetic background of GPx1/2-DKO mice is known to have a strong influence on disease course. Here, we show that the genetic background also has a strong impact on cecal microbiota. In fact, the genetic background of the inbred mice has the strongest impact on the gut microbiota compared to diet and GPx genotype. Since the mouse colonies were housed on the same cage rack, it is unlikely that the different microbiota come from physical separation. Since B6 and 129 strains may have different innate immune responses, which can modulate microflora community [2, 13], our result provides an evidence to support the notion that host genetics can affect gut microbiota. A strong genetic effect on microflora has been observed in humans from studies of monozygotic and dizygotic twins versus cohabitating, unrelated individuals [29–31].

Since Paneth cells in the mouse ileum express multiple genes encoding for antimicrobial peptides including *α*-defensin-related cryptidins and the mouse-specific cryptidin-related sequences (Defcr-rs) [32], it is possible that the quantity and/or types of antimicrobial peptides affect gut microbiota composition (our unpublished observation from Agilent 44K Mouse Expression Array analysis). The Defcr-rs gene subfamily that codes for several cysteine-rich-sequence-4C (CRS4C) peptides is unique to mice and these CRS4C peptides also have bactericidal activity [32]. We have found that 129 strain mice have elevated *Defcr-rs2 *(encoding for CRS4C-1)*, -rs10 *(CRS4C-4)* and -rs12 *(CRS3C-5) gene expression in the ileum of both DKO and WT mice, when B6 has low or undetectable expression of these genes. Shanahan et al. reported that *Defcr-rs2 and -rs10* genes were highly expressed in SAMP1/YitFc mice (which had spontaneous ileitis) and barely detectable in B6 strain. Since both 129 strain mice and SAMP1/YitFc mice with high expression of some of the *Defcr-rs* genes are prone to inflammation, it was postulated that the cysteine-rich CRS4C peptides may also affect cellular homeostasis such as affecting autophagy, unfolded protein response, and apoptosis. Because of the complex nature of the defensin gene family in mice, we plan to explore this issue further and present it in a comprehensive manner in the future.

Although the ARISA profile has limited resolution, we were able to identify 9 species that belong to 3 bacteria phyla, *Firmicutes*, *Bacteroides*, and *Proteobacteri*, commonly present in human gut. From our small list of bacteria, we have identified *E. coli* as a potential harmful bacteria species, where overgrowth is associated with 129 but not B6 mice. With the ARISA primers,* E. coli* produces a 442 bp PCR product, which is present in the 129 strain and absent in B6 mice. In the 129 strain, this 442 bp ribotype is more abundant in the DKO than that control non-DKO mice, with the only exception of inulin-fed non-DKO mice. Furthermore, this 442 bp PCR product is more prominent in the sick than well 129 N5 DKO mice. Although the 442 bp ribotype is also matched to *Shigella *and *Salmonella* in the *Proteobacteri *bacteria phyla by sequence analysis, our isolation of *E. coli* species from several 129 DKO mice supports the *E. coli* identity of this 442 bp DNA PCR product. The sick 129 DKO mice also have a more prominent 517 bp DNA also produced by our cultured putative *E. coli *clones as well as from a laboratory strain of *E. coli,* DH5*α*. Detection of the 442 bp but not the 517 bp PCR products may be because the larger product is not as efficiently amplified as the shorter one when *E. coli* DNA is present in a mixture of other bacteria DNA. The results shown in the ARISA panels coupled with counts from diluted cecal contents on LB plates suggest that detection of the 442 bp ribotype peak required 1 × 10^8^-1 × 10^9^  CFU/g. Since most of the well mice, B6 or 129, have counts less than 2 × 10^7^ CFU/g, the counts obtained for sick 129 DKO mice appear to represent an overgrowth of *E. coli*. Enteropathic *E. coli* has been linked to Crohn's disease and *Salmonella* to enterocolitis by opportunistic invasion of inflamed areas [7, 33–37]. These bacteria may create the opportunity for overcoming colonization resistance by eliciting an inflammatory response and then invading the inflamed region [37]. 

The DKO mice are more resistant to dietary impact on microbiota profiles than control mice. This result is consistent with the notion that inflammation in DKO mice diminishes bacteria diversification relative to that in the control mice, which was reported in human IBD patients [3, 4]. The limitations on bacterial diversity in DKO mice can explain why diet alters ARISA profiles only in B6 WT but not in B6 DKO mice. Although 129 N5 mice on either AIN or LabDiet have rather similar profiles, DKO mice on these two diets have higher similarity index than non-DKO mice on these two diets. Non-DKO mice were used because they were littermates of DKO mice, thus shared the same bedding but did not have gut pathology. 

Supplementation of yeast to the AIN diet changed bacteria profile dramatically in 129 N5 DKO mice. Yeast produced a prominent 317 bp ribotype matched to *Lactobacillus animalis* or *L. murinus* in *Firmicutes* phylum. Although the *Lactobacillus sp.* have been used as probiotics, they can also induce reactive oxygen species (ROS) from macrophages [38]. This pro-oxidant property of these bacteria may blunt their probiotic activity in the DKO mice. This may explain why the yeast-AIN diet increased morbidity and diarrhea, but not colon inflammation, in 129 N5 DKO mice. It is unclear why the LabDiet (which has 1% brewer's yeast) did not produce this 317 bp ribotype in both 129 N5 DKO and non-DKO mice. Perhaps other ingredients in the LabDiet have masked or countered the effect of yeast. IBD patients tend to have high antibody titers against yeast [39], and mannan-containing yeast-wall components are strong activators for macrophages and neutrophils [40]. However, our result does not support the proinflammatory activity of yeast.

Inulin is a prebiotic, which has been used to manage IBD by changing gut microbiota [1, 26]. Dietary supplementation of inulin increases the number of *Bifidobacteria* in humans and animals [41, 42]. Interestingly, supplementing inulin to AIN diet has an opposing effect on microflora between 129 N10 DKO and control mice. Inulin increases bacteria diversity in DKO but decreases diversity in control mice. Consequently, the diverse ARISA profiles are more distinct from each other, when the converged profiles become similar. Most disappointedly, inulin did not eliminate the 442 bp, likely from *E. coli* colonization. This may explain the lack of anti-inflammatory activity of inulin in 129 DKO mice.

The impact of the GPx1/2-DKO construct on microbial diversity is likely due to increases in reactive oxygen species and activation of innate immune response. We have shown that the DKO intestine has higher levels of lipid peroxidation and myeloperoxidase/lactoperoxidase activity compared to non-DKO control mice [10]. This increased oxidative stress may explain the elevated apoptotic cell death in the crypt epithelium accompanied by degranulation of Paneth cells and mucin depletion [11]. The primary function of enteric defensins is thought to be a regulator of intestinal microbiota [43]. Mucin excretion can inhibit bacterial adhesion [44]. Conceivably, overexpression or depletion of these antimicrobial molecules can alter luminal microflora.

## 5. Conclusions

In summary, using a nonculture ARISA we examined the effect of mouse strain background, diet and DKO genotype on the gut microbiota. We found that B6 versus 129 strain background has the greatest overall impact on the microbiota with similarity indexes from 0.26 to 0.12 when matched for diet and GPx status. GPx status did not affect the strain-based dissimilarity. In comparing AIN and LabDiet, DKO mice were more resistant to change than the non-DKO or WT mice in both B6 and 129 strains. This may be due to inflammation in the DKO gut, which has restricted microflora diversity. However, supplementing yeast and inulin to AIN diet greatly altered microflora profiles in the DKO mice. We have identified a 442 bp DNA from *E. coli*, as a proinflammatory bacteria associated with 129 strain mice especially with the DKO genotype.

## Figures and Tables

**Table 1:** Diet compositions.

Contents	LabDiet^1^	LabDiet	AIN^1^	AIN	AIN + inulin	Brewer's	Yeast (BY)^2^
	5061	5062	5% CO	10% CO	5–10% CO	1% BY	10% BY
Protein	Mixed	Mixed	Casein	Casein	Casein	Mixed	Mixed
(%)^3^	(23.4)	(20.5)	(23.4)	(17.7)	(20–26.5)	(17.8)	(17.7)
Yeast	Brewer's	Brewer's	—^4^	—	—	Brewer's	Brewer's
(% wt)	(1)	(1)				(1)	(10)
DL-Methionine, %	(0.43)	(0.48)	(0.3)	(0.3)	(0.3)	(0.3)	(0.3)
Fat	Mixed	Mixed	Corn oil	Corn oil	Corn oil	Corn oil	Corn oil
(%)	(5.5)	(9.6)	(5.0)	(10.0)	(5–10)	(10.3)	(10.7)
Sucrose, %	(3.7)	(0.4)	(50)	(39)	(5.0–38.4)	(38)	(35)
Other CHO^5^	Mixed	Mixed	Corn^5^	Corn	Corn	Corn	Corn
(%)	(56)	(53)	(5)	(15)	(27.5–15.5)	(15)	(15)
Cellulose (fiber), %	(5.3)	(2.7)	(18.4)	(11.5)	(13.4–6.5)	(11.5)	(11.5)

Kcal/g	3.3	3.8	3.1	3.8	3.1–3.8	3.8	3.8

^1^LabDiets contained crude ingredients and micronutrients similar to AIN-76A (http://www.testdiet.com/). Other diets were defined diets containing AIN-76A mineral and vitamin mix. All defined diets contained ~0.002% ethoxyquin as an antioxidant, and had an adequate amount (0.2%) of choline bitartrate or choline chloride.

^2^Protein, carbohydrate, and fat in Brewer's yeast are not included in other contents.

^3^All % content is by weight.

^4^A dash means not present.

^5^CHO means carbohydrate, and Corn means corn starch.

**Table 2:** Identification of ARISA peak DNA.

Base pair	Top BLAST hits of ARISA peak DNA^1^	Phylum	Matched from bacterial culture^2^
293^1^	*Lactobacillus animalis/murinus*	*Firmicutes*	*Lactobacillus sp.* ^3^
300		*Firmicutes*	*Enterococcus gallinarum*
317^1^	*Lactobacillus animalis/murinus*	*Firmicutes*	
350		*Firmicutes*	*Enterococcus faecalis*
380		*Firmicutes*	*Staphylococcus hemolyt./saprophy.*
385–390^1,3^	*Lactobacillus sp.*	*Firmicutes*	*Lactobacillus sp.^3^*
	Uncultured bacteria CD^4^	*Bacteroides sp.*	
400		*Firmicutes*	*Staphylococcus simulins*
421^1^	*Bacteroides sp.*	*Bacteroidetes*	
	Uncultured bacteria CD^4^	*Bacteroides sp.*	
442^1^	*Escherichia coli, Shigella, Salmonella*	**γ*-Proteobacteri*	*E. coli^3^*
480		*Firmicutes*	*Lactobacillus sp.^3^*
517^1^	*E. coli, Shigella, Salmonella*	**γ*-Proteobacteri*	*E. coli^3^*
	Uncultured bacteria CD^4^	*Bacteroides sp*.	
586^1^	*Dysgonomonas wimpennyi*	*Bacteroidetes*	
710^1^	*E. coli APEC, Shigella, Salmonella*	**γ*-Proteobacteria*	
792^1^	*Helicobacter ganmani*	**ε*-Proteobacteria*	
800		*Actinobacteria*	*Bifidobacterium sp.*

^1^Eight prominent ARISA peak DNAs plus the 710 bp DNAs were cloned into plasmid and sequenced. The identity of the ARISA fragments was determined using a Basic Local Alignment Search Tool (BLAST;   http://www.ncbi.nih.gov/).

^2^The ARISA peak DNA size was matched to the PCR product of cultured bacterial DNA from mouse cecal content using the same primers.

^3^Due to the clustering of DNA products in the 385–390 bp and limitation of the cloning/sequencing vector, the specific size of the clones cannot be determined. Because multiple ribosomal intergenic spacers exist in each bacterium, single *E. coli* colonies produced amplicons at *∼*442, *∼*517, and *∼*710 bp and single colonies of *Lactobacillus *produced amplicons at *∼*293, 385–390, and *∼*480 bp.

^4^DNA sequences obtained from at least one clone each of ARISA DNA at 385–390, 421, and 517 bp were matched to an uncultured bacteria CD, LMOACA3ZDO3RM1 clone, submitted by Manichanh et al. obtained from the fecal sample of a Crohn's disease patient [27].
